# NMR Analysis of Poly(Lactic Acid) via Statistical Models

**DOI:** 10.3390/polym11040725

**Published:** 2019-04-19

**Authors:** Koto Suganuma, Tetsuo Asakura, Miyuki Oshimura, Tomohiro Hirano, Koichi Ute, H. N. Cheng

**Affiliations:** 1 Material Analysis Research Center, Teijin Ltd, Hino, Tokyo 191-8512, Japan; 2 Department of Biotechnology, Tokyo University of Agriculture and Technology, Koganei, Tokyo 184-8588, Japan; asakura@cc.tuat.ac.jp; 3 Department of Applied Chemistry, Tokushima University, 2-1 Minamijosanjima, Tokushima 770-8506, Japan; oshimura@tokushima-u.ac.jp (M.O.); hirano@tokushima-u.ac.jp (T.H.); ute@tokushima-u.ac.jp (K.U.); 4 Southern Regional Research Center, USDA Agricultural Research Service, 1100 Robert E. Lee Blvd., New Orleans, LA 70124, USA; hn.cheng@usda.gov

**Keywords:** Bernoullian, NMR, poly(lactic acid), statistical model, stereosequences, tacticity

## Abstract

The physical properties of poly(lactic acid) (PLA) are influenced by its stereoregularity and stereosequence distribution, and its polymer stereochemistry can be effectively studied by NMR spectroscopy. In previously published NMR studies of PLA tacticity, the NMR data were fitted to pair-addition Bernoullian models. In this work, we prepared several PLA samples with a tin catalyst at different L,L-lactide and D,D-lactide ratios. Upon analysis of the tetrad intensities with the pair-addition Bernoullian model, we found substantial deviations between observed and calculated intensities due to the presence of transesterification and racemization during the polymerization processes. We formulated a two-state (pair-addition Bernoullian and single-addition Bernoullian) model, and it gave a better fit to the observed data. The use of the two-state model provides a quantitative measure of the extent of transesterification and racemization, and potentially yields useful information on the polymerization mechanism.

## 1. Introduction

Poly(lactic acid) (PLA) is a biodegradable and thermoplastic aliphatic polyester derived from renewable resources, such as corn, cassava, and sugarcane. As a popular bioplastic, it has favorable physical and environmental properties [1,2,3] and is used in many industrial, biomedical, therapeutic, and pharmaceutical applications such as drug delivery systems, protein encapsulation and delivery, tissue engineering, sutures, and prostheses [4,5,6,7]. A major determinant of the physical and end-use properties of PLA is polymer stereochemistry—thus, racemic PLA is known to have a low glass transition temperature (*T*_g_), but poly(L-lactic acid) (PLLA) and poly(D-lactic acid) (PDLA) can form a stereo-complex that has a higher *T*_g_ and improved mechanical properties [7,8]. A better understanding of PLA stereochemistry is therefore useful both in fundamental studies of structure/property relationships and in commercial product development.

NMR is known to be one of the best techniques for the analysis of polymer stereochemistry and tacticity [9,10]. Thus, there have been a large number of NMR tacticity studies of PLA (mostly in CDCl_3_) at the tetrad level for CH protons and carbon, and partially at the hexad level for carbonyl carbon [11,12,13,14,15,16,17,18,19,20,21,22,23,24]. The assignments were assisted with quantum chemical calculations of chemical shifts [25,26]. Recently, pyridine-d_5_ has been found to have a notable solvent effect on the NMR spectra of PLA due to the pyridine ring current effect, and as a result, better resolved peaks of the methyl proton have been successfully obtained and partially assigned [27].

Statistical models (also known as reaction probability models) have been used extensively in polymer studies [28,29,30]. They provide fundamental understanding of polymerization processes and serve as a theoretical framework whereby NMR, molecular weight, fractionation, and polymerization kinetics data can be analyzed in a rational manner. In many cases the models can assist in NMR spectral assignments and help to interpret the spectral intensities. Sometimes they reveal information on the mechanisms of initiation and propagation [28,29,30,31]. For PLA, if the starting material is lactide (the cyclic dimer of lactic acid), the simplest model is the pair-addition Bernoullian model that takes into account the configuration of lactide. There are some variations on this model, depending on the details of the formulation used [19,20,21,22,23,24]. Several publications took different versions of this model and applied them to NMR data [19,20,21,22,23,24]. The fit was usually satisfactory when no transesterification or racemization was present. However, when transesterification and racemization occurred, deviations from the pair-addition Bernoullian models were found [20,22,23].

In this work, we took several PLA samples synthesized with a tin catalyst from different ratios of L,L-lactide and D,D-lactide and analyzed the NMR tacticity. Indeed, the data showed noticeable departure from the pair-addition Bernoullian model. We introduced a two-state model, consisting of both the pair-addition Bernoullian and single-addition Bernoullian models. The inclusion of the single-addition Bernoullian model permitted a quantitative evaluation of the role of transesterification and racemization. This two-state model was found to fit the data reasonably well. As far as we know, this is the first report of the use of a two-state model for the NMR analysis of PLA.

## 2. Materials and Methods

### 2.1. Materials

The L,L-lactide and *rac*-lactide (Tokyo Chemical Industry Co., Ltd., Tokyo, Japan) were purified by recrystallization from hexane/toluene. Tin (II) bis(2-ethylhexanoate) (Sn(Oct)_2_, Sigma-Aldrich, Tokyo, Japan), chloroform, and methanol (Kanto Chemical Co. Inc., Tokyo, Japan) were used as received. The NMR solvent (CDCl_3_) was obtained from Fujifilm Wako Pure Chemical Corporation, Osaka, Japan.

### 2.2. Synthesis of PLA

**Scheme 1:**
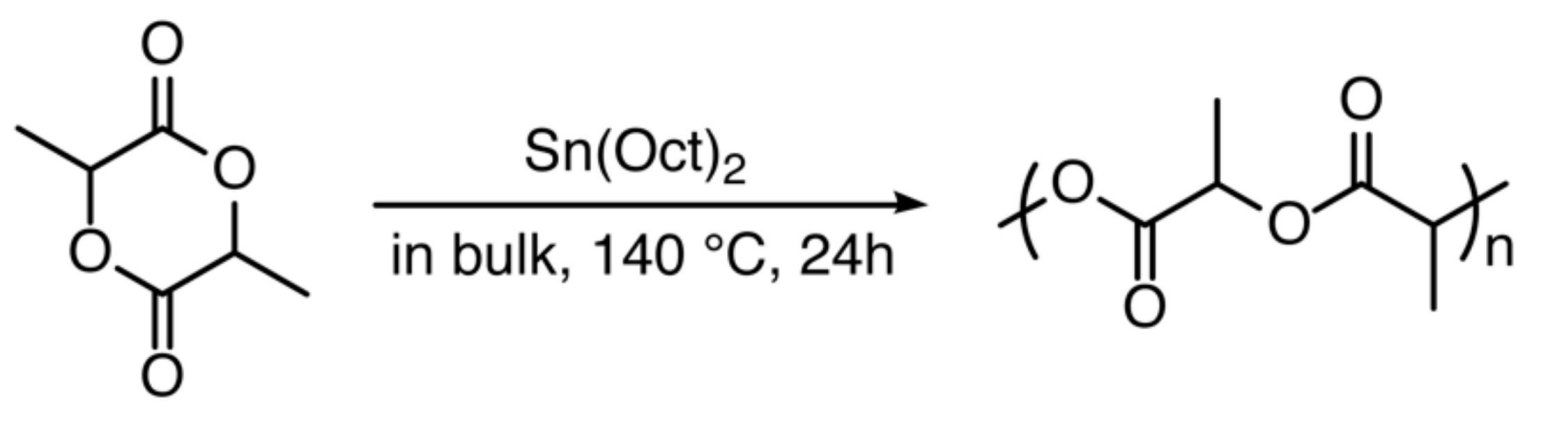
Conversion of lactides into PLA

Ring-opening polymerization of lactides (1.4 g, 10 mmol) with tin (II) bis(2-ethylhexanoate) (0.04 g, 0.1 mmol) was carried out in bulk at 140 °C for 24 h [27] (Scheme 1). The product was dissolved in chloroform and poured into an excess amount of methanol. The precipitated polymer was collected by centrifugation or suction filtration and then dried in vacuo. The molecular weights and molecular weight distribution were determined by size-exclusion chromatography (SEC)—the chromatograph was calibrated with standard polystyrene samples. SEC was performed on an HLC-8220 chromatograph (Tosoh, Tokyo, Japan) equipped with TSKgel columns (SuperHM-M (6.5 mm inner diameter × 150 mm long) and SuperHM-H (6.5 mm inner diameter × 150 mm long) (Tosoh, Tokyo, Japan). *N*,*N*-dimethylformamide (DMF) containing 10 mmol L^−1^ LiBr was used as the eluent at 40 °C at a flow rate of 0.35 mL min^−1^. The initial polymer concentration was set at 1.0 mg mL^−1^.

### 2.3. NMR Analysis

The spectra were acquired at ambient temperature on an ECA-600 spectrometer (JEOL Ltd, Tokyo, Japan) operating at 600 MHz (^1^H) or 150 MHz (^13^C). The polymers were dissolved in CDCl_3_ at a concentration of 2% (^1^H) or 15% (^13^C) (*w*/*v*). Tetramethylsilane (TMS) was used as an internal chemical shift reference. The ^1^H NMR spectra were obtained with a digital resolution of 0.36 Hz/point with 32 K data points together with a 45° flip angle and a 4 s pulse delay. For ^1^H homonuclear decoupling, the irradiation position was set to 1.57 ppm and the power was optimized to achieve the best results. The ^13^C NMR spectra were obtained with ^1^H broadband decoupling and a digital resolution of 1.44 Hz/point with 32 K data points together with a 45° flip angle and a 2 s pulse delay.

An analysis of the NMR intensities via different statistical models was conducted with a custom-written QuickBASIC program called MIXCO.PLA. The parameters were varied via a simplex algorithm by minimizing the mean deviation between the observed and calculated intensities. The flow chart and logic of the program were similar to the NMR tacticity and sequence analysis programs reported earlier [32,33]. Interested readers may write to the corresponding authors for a copy of the program.

## 3. Results and Discussion

### 3.1. Preparation of the PLA Samples

Ring-opening polymerization of lactides with different LL/DD ratios was performed to prepare PLA samples with different tacticities (Table 1). The LL/DD ratios were changed from 50/50 to 90/10 by mixing *rac*-lactide with L,L-lactide.

These five samples were made with the same catalyst under the same experimental conditions. They showed similar reaction yields and molecular weights. Thus, these samples constituted a good data set for NMR analysis.

### 3.2. NMR Data and Assignments

**Figure 1:**
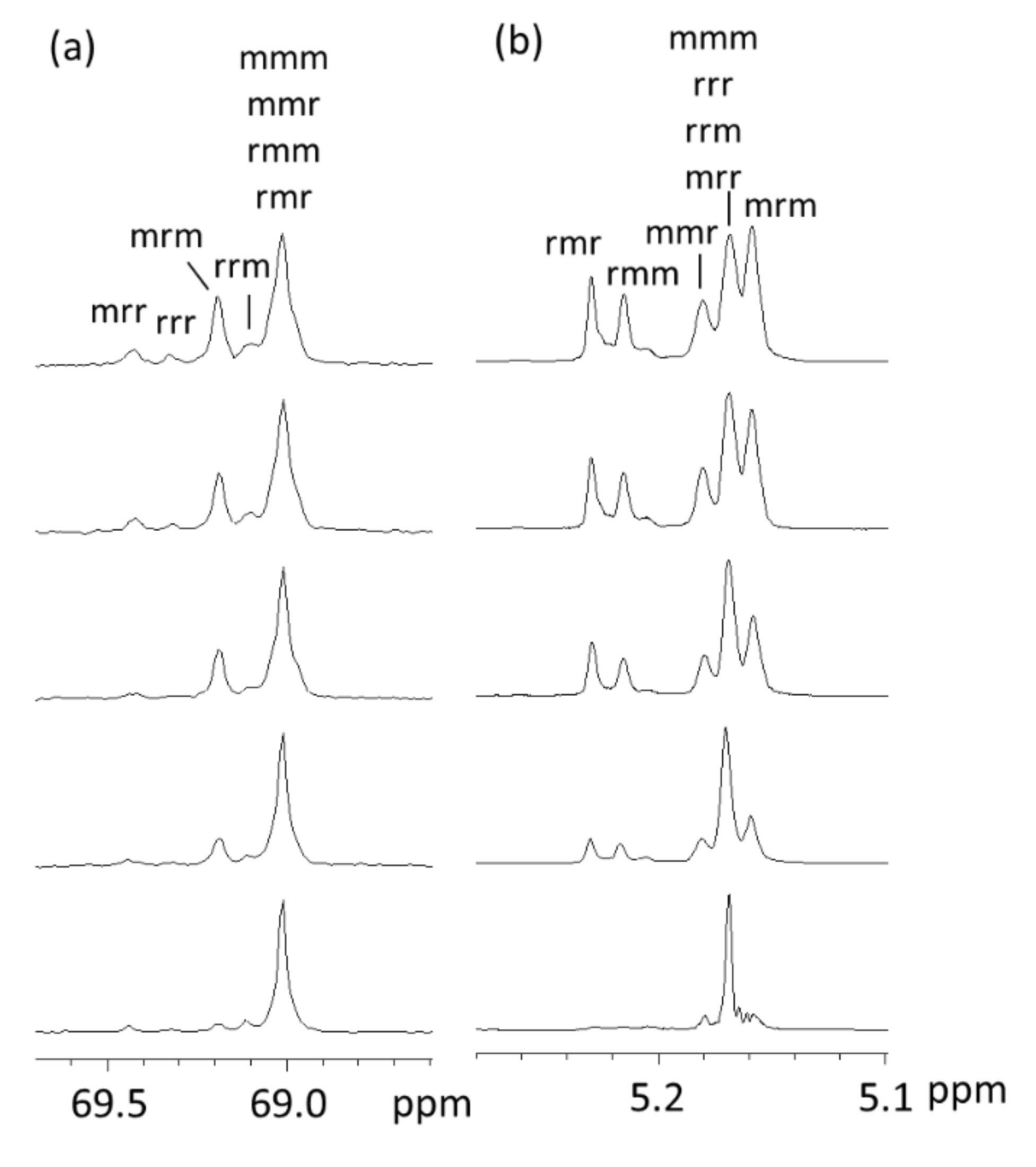
(**a**) ^13^C and (**b**) ^1^H NMR spectra of the CH groups of the PLA samples (from the top to the bottom: LL/DD = 50/50, 60/40, 70/30, 80/20, 90/10).

Figure 1 shows the ^13^C and ^1^H NMR spectra of the methine region of the PLA samples. The splitting of the methine proton resonance caused by coupling to the methyl protons was removed by homonuclear decoupling [15,19,34]. The signals were assigned at the tetrad level as summarized in Table 2, according to the literature [11,12,13,14,15,16,17,18,19,20,21,22,23,24]. Four racemo r-centered tetrads (mrr, rrr, mrm, rrm) can be determined from the integral intensities of the CH carbon, whereas three meso m-centered tetrads (rmr, rmm, mmr) can be determined from those of the CH proton. Therefore, the fraction of the mmm tetrad was calculated by subtracting the sum of the other seven tetrad fractions from 100%.

### 3.3. Analysis of NMR Data

In order to derive more information from tacticity, we attempted to fit the NMR data to an appropriate statistical model for polymerization. The configurations in the lactide monomers are retained through ring-opening polymerization, unless side reactions, such as transesterification and racemization, occur. In other words, the lactides are incorporated as m diads in the resulting polymer. Therefore, the pair-addition Bernoullian (PAB) model can be used. Different variations of this model have been previously published [19,20,21,22,23,24]. For convenience, we used Price’s formalism for tacticity [35,36] and adopted the tetrad expressions reported previously by Schindler and Harper [24] and Chabot et al. [23]. These expressions are summarized in column 2 of Table 3.

The intensities calculated with the PAB model are given in Table 4, together with the observed ones. As p_2_ + q_2_ = 1, there was only one independent parameter (p_2_) for this model. For each sample, the monomer feed fractions were first applied as the p_2_ values, such as 0.50, 0.60, 0.70, 0.80 and 0.90, and the calculated values were summarized as *I*_calc1_. The mean deviations (MDs) obtained with all these fittings appeared to be large. The p_2_ value was then allowed to vary in order to obtain the least MDs between observed and calculated tetrad intensities. The calculated values were summarized as *I*_calc2_. In the case of sample 50/50, the fitted value was the same as the feed value of 0.50. In all cases, the calculated intensities for mrr, rrm, and rrr tetrads were zero, because it was impossible to form these stereosequences by the polymerization of mixtures of L,L- and D,D-lactides. In general, the fitted p_2_ values were rather close to the feed p_2_ values. Only sample 70/30 showed a somewhat greater variation between the theoretical and calculated values of p_2_. Nonetheless, in all five samples the MD values were rather large, indicating a relatively poor fit.

It is known that transesterification and racemization occur to some extent in PLA polymerization catalyzed with tin alkanoate [7,20,22,23]. An improved analytical methodology is then needed to account for this complication. The proposition in this work is to employ a two-state model, involving both pair-addition Bernoullian (PAB) and single-addition Bernoullian (SAB) models. The SAB component then represents the effect of transesterification and racemization. The theoretical expressions for SAB correspond to the tacticity in Price’s formalism and have been previously reported [35,36]. They have also been separately reported with respect to PLA tacticity [23]. These expressions are summarized in the third column of Table 3. The expressions for the two-state (PAB/SAB) model are then the weighted sums of the corresponding expressions for the PAB and the SAB models, as shown in column 4 of Table 3. As p_2_ + q_2_ = 1, p_1_ + q_1_ = 1, f_1_ + f_2_ = 1, there are three independent parameters for this two-state model (p_2_, f_2_, p_1_).

The observed PLA tetrad data were fitted to the two-state (PAB/SAB) model using the same fitting program, and the results are shown in Table 5. Initially, all p_1_ values (in the SAB component) were set around 0.5 to represent complete racemization (*I*_calc1_ in Table 5) and the other two parameters (p_2_, f_2_) were allowed to vary, but the MDs, although better than the deviations for the PAB model, still seemed large.

By permitting all three parameters to vary, we obtained lower MDs (*I*_calc2_ in Table 5). The only exception was sample 50/50, where the result with p_1_ = p_2_ = 0.5 was the best fit obtained. The results appeared to be more satisfactory with MDs of 1.5 or less. It may be noted that the fitted p_2_ values matched well with the expected feed LL/(LL + DD) ratio. Interestingly, in all results, p_2_ and p_1_ were approximately equal. Thus, for this data set, transesterification and racemization appeared to preserve the configuration. The amount of the SAB content (f_1_) was roughly 30%, with a lower value for sample 70/30 and a higher value for sample 90/10. 

Although the fitted results in Table 5 appear promising, these results should be considered tentative. The main intent of this work was to establish the analytical methodology. As we analyze more PLA samples in the future, more definite trends will hopefully emerge.

## 4. Conclusions

In the NMR analysis of polymers, statistical models are useful in providing a theoretical framework for analysis, checking the consistency of results, and deriving information on polymerization mechanisms. In this work, NMR analysis and statistical model fitting were applied to several PLA samples made from mixtures of L,L- and D,D-lactides with a tin catalyst. The NMR tetrads were first fitted to the pair-addition Bernoullian model and found to be unsatisfactory due to the presence of transesterification and racemization. Thus, a two-state (pair-addition Bernoullian and single-addition Bernoullian) model was developed as an alternative model. This model was found to provide improved fit with the NMR tetrad data. As the NMR tetrad (and hexad) data of more PLA samples become available, the use of two-state and other more refined statistical models for the analysis of NMR data is expected to provide further information on polymer structures and reaction mechanisms.

## Figures and Tables

**Table 1:** Bulk polymerization of lactide with Sn(Oct)_2_ at 140 °C for 24 h.

Sample Code	Lactide in Feed		Yield/%	*M*_n_ × 10^−3^	*M*_w_/*M*_n_
	L,L	D,D			
50/50	50	50	74	13.0	1.56
60/40	60	40	62	13.2	1.66
70/30	70	30	69	14.5	1.66
80/20	80	20	70	13.5	1.68
90/10	90	10	72	11.2	2.96

**Table 2:** The chemical shifts of the tetrad peaks of poly(lactic acid) (PLA) methine groups.

Tetrad	mrr	rrr	mrm	rrm	mmm/mmr/rmm/rmr
^13^C shift (ppm)	69.42	69.32	69.19	69.09	69.02
**Tetrad**	**rmr**	**rmm**	**mmr**	**mmm/rrr/rrm/mrr**	**mrm**
^1^H shift (ppm)	5.23	5.22	5.18	5.17	5.16

**Table 3:** Theoretical expressions for tetrad fractions in the various statistical models used for the NMR analysis of the polymerization of mixtures of L,L- and D,D-lactides.

Tetrad	Pair-Addition Bernoullian Model ^a^	Single-Addition Bernoullian Model ^b^	Two-State (Pair-Addition + Single-Addition Bernoullian) Model ^c^
mmm	(p_2_^2^ + q_2_^2^ + p_2_^3^ + q_2_^3^)/2	p_1_^4^ + q_1_^4^	f_2_[(p_2_^2^ + q_2_^2^ + p_2_^3^ + q_2_^3^)/2] + f_1_[p_1_^4^ + q_1_^4^]
mrm	p_2_q_2_	2p_1_^2^q_1_^2^	f_2_ p_2_q_2_ + f_1_ [2p_1_^2^q_1_^2^]
mmr	p_2_q_2_/2	p_1_^3^q_1_ + p_1_q_1_^3^	f_2_ p_2_q_2_/2 + f_1_ [p_1_^3^q_1_ + p_1_q_1_^3^]
rmm	p_2_q_2_/2	p_1_^3^q_1_ + p_1_q_1_^3^	f_2_ p_2_q_2_/2 + f_1_ [p_1_^3^q_1_ + p_1_q_1_^3^]
rmr	p_2_q_2_/2	2p_1_^2^q_1_^2^	f_2_ p_2_q_2_/2 + f_1_ [2p_1_^2^q_1_^2^]
rrm	0	p_1_^3^q_1_ + p_1_q_1_^3^	f_1_ [p_1_^3^q_1_ + p_1_q_1_^3^]
mrr	0	p_1_^3^q_1_ + p_1_q_1_^3^	f_1_ [p_1_^3^q_1_ + p_1_q_1_^3^]
rrr	0	2p_1_^2^q_1_^2^	f_1_ [2p_1_^2^q_1_^2^]

^a^ The pair-addition reaction probabilities p_2_ = LL/(LL + DD) and q_2_ = DD/(LL + DD). ^b^ The single-addition reaction probabilities p_1_ = L/(L + D) and q_1_ = D/(L + D). ^c^ The fractions of single-addition and pair-addition are f_1_ and f_2_, respectively, where f_1_ + f_2_ = 1.

**Table 4:** NMR intensities (%) of tetrad fractions observed and calculated with the pair-addition Bernoullian model. ^a^

Tetrad	Sample 50/50		Sample 60/40			Sample 70/30			Sample 80/20			Sample 90/10		
	*I*_obsd_	*I*_calc_	*I*_obsd_	*I*_calc1_	*I*_calc2_	*I*_obsd_	*I*_calc1_	*I*_calc2_	*I*_obsd_	*I*_calc1_	*I*_calc2_	*I*_obsd_	*I*_calc1_	*I*_calc2_
mmm	26.3	37.5	30.0	40.0	38.0	39.9	47.5	40.1	49.4	60	57.7	71.5	77.5	71.5
mrm	22.3	25.0	20.7	24.0	24.8	21.4	21.0	24.0	13.5	16	16.9	5.2	9.0	11.4
mmr	13.4	12.5	13.6	12.0	12.4	11.3	10.5	12.0	7.4	8	8.5	6.9	4.5	5.7
rmm	13.0	12.5	12.5	12.0	12.4	10.9	10.5	12.0	7.7	8	8.5	3.5	4.5	5.7
rmr	12.9	12.5	12.3	12.0	12.4	12.0	10.5	12.0	7.8	8	8.5	2.5	4.5	5.7
mrr	6.5	0	6.0	0	0	2.5	0	0	3.9	0	0	3.9	0	0
rrm	3.6	0	2.7	0	0	1.5	0	0	9.1	0	0	4.7	0	0
rrr	2.0	0	2.4	0	0	0.7	0	0	1.2	0	0	1.8	0	0
MD ^b^		3.5		3.4	3.1		1.9	1.2		3.6	3.5		3.2	2.9
p_2_		0.50		0.60	0.55		0.70	0.60		0.8	0.78		0.90	0.87

^a^ Tetrad intensities were normalized to 100%. ^b^ Values were obtained via the program MIXCO.PLA. Mean deviations (MDs) between observed and fitted values.

**Table 5:** NMR intensities (%) of tetrad fractions observed and calculated with the two-state pair-addition Bernoullian (PAB)/ single-addition Bernoullian (SAB) model. ^a^

Tetrad	Sample 50/50		Sample 60/40			Sample 70/30			Sample 80/20			Sample 90/10		
	*I*_obsd_	*I*_calc_	*I*_obsd_	*I*_calc1_	*I*_calc2_	*I*_obsd_	*I*_calc1_	*I*_calc2_	*I*_obsd_	*I*_calc1_	*I*_calc2_	*I*_obsd_	*I*_calc1_	*I*_calc2_
mmm	26.3	29.9	30.0	30.0	30.0	39.9	42.6	39.9	49.4	53.9	49.4	71.5	71.5	71.5
mrm	22.3	21.2	20.7	19.2	20.6	21.4	20.0	21.3	13.5	14.8	13.5	5.2	10.7	5.2
mmr	13.4	12.5	13.6	12.2	12.4	11.3	11.0	11.6	7.4	9.5	9.6	6.9	6.9	6.1
rmm	13.0	12.5	12.5	12.2	12.4	10.9	11.0	11.6	7.7	9.5	9.6	3.5	6.9	6.1
rmr	12.9	12.5	12.3	12.2	12.3	12.0	11.0	11.3	7.8	9.5	7.8	2.5	6.9	3.1
mrr	6.5	3.8	6.0	5.2	4.1	2.5	2.0	1.5	3.9	4.1	3.8	3.9	3.1	3.9
rrm	3.6	3.8	2.7	5.2	4.1	1.5	2.0	1.5	9.1	4.1	3.8	4.7	3.1	3.9
rrr	2.0	3.8	2.4	5.2	4.1	0.7	2.0	1.2	1.2	4.1	2.0	1.8	3.1	0.9
MD ^b^		1.5		1.2	0.8		1.0	0.4		2.4	1.3		2.1	0.7
p_2_		0.50		0.60	0.55		0.69	0.65		0.80	0.77		0.89	0.90
f_2_		0.70		0.59	0.67		0.84	0.88		0.67	0.66		0.75	0.48
p_1_		0.50		0.51	0.55		0.50	0.66		0.50	0.78		0.50	0.9
f_1_^c^		0.30		0.41	0.33		0.16	0.12		0.33	0.34		0.25	0.52

^a^ Tetrad intensities were normalized to 100%. Values were obtained via the program MIXCO.PLA. ^b^ MDs between observed and fitted values. ^c^ The value of f_1_ was not fitted—it was computed from f_2,_ where f_1_ = 1−f_2_.
